# Residual biliary intraepithelial neoplasia without malignant transformation at resection margin for perihilar cholangiocarcinoma does not require expanded resection: a dual center retrospective study

**DOI:** 10.1186/s12957-024-03395-5

**Published:** 2024-06-21

**Authors:** Zeliang Xu, Xiaoyi Fan, Chengcheng Zhang, Yuancheng Li, Di Jiang, Feng Hu, Bi Pan, Yixian Huang, Leida Zhang, Wan Yee Lau, Xingchao Liu, Zhiyu Chen

**Affiliations:** 1 Department of Hepatobiliary Surgery, Southwest Hospital, Third Military Medical University (Army Medical University), Chongqing, China; 2 Department of Oncology and Southwest Cancer Center, Southwest Hospital, Army Medical University (Third Military Medical University), Chongqing, China; 3 Faculty of Medicine, the Chinese University of Hong Kong, Shatin, New Territories, Hong Kong SAR China; 4 Department of Hepatobiliary Surgery, School of Medicine, Sichuan Provincial People’s Hospital, University of Electronic Science and Technology of China, No. 32, Qingyang District, Chengdu, 610072 China

**Keywords:** Perihilar Cholangiocarcinoma, Surgical resection, Intraoperative frozen pathology, Biliary intraepithelial neoplasia

## Abstract

**Background:**

Additional resection for invasive cancer at perihilar cholangiocarcinoma (pCCA) resection margins has become a consensus. However, controversy still exists regarding whether additional resection is necessary for residual biliary intraepithelial neoplasia (BilIN).

**Method:**

Consecutive patients with pCCA from two hospitals were enrolled. The incidence and pattern of resection margin BilIN were summarized. Prognosis between patients with negative margins (R0) and BilIN margins were analyzed. Cox regression with a forest plot was used to identify independent risk factors associated with overall survival (OS) and recurrence-free survival (RFS). Subgroup analysis was performed based on BilIN features and tumor characteristics.

**Results:**

306 pCCA patients receiving curative resection were included. 255 had R0 margins and 51 had BilIN margins. There was no significant difference in OS (*P* = 0.264) or RFS (*P* = 0.149) between the two group. Specifically, 19 patients with BilIN at distal bile ducts and 32 at proximal bile ducts. 42 patients showed low-grade BilIN, and 9 showed high-grade. Further analysis revealed no significant difference in long-term survival between different locations (*P* = 0.354), or between different grades (*P* = 0.772). Portal vein invasion, poor differentiation and lymph node metastasis were considered independent risk factors for OS and RFS, while BilIN was not. Subgroup analysis showed no significant difference in long-term survival between the lymph node metastasis subgroup, or between the portal vein invasion subgroup.

**Conclusion:**

For pCCA patients underwent curative resection, residual BilIN at resection margin is acceptable. Additional resection is not necessary for such patients to achieve absolute R0 margin.

## Introduction

Cholangiocarcinoma, apart from hepatocellular carcinoma, is the most common malignant tumor of the liver. It can sub-classified as intrahepatic, distal and perihilar cholangiocarcinoma (pCCA), with pCCA being the most common type characterized by aggressive malignancy and an unfavorable prognosis [1–3]. Curative surgical resection is the only treatment that can offer a cure for those patients, with a 5-year survival rate reported to range from 14 to 45% [4–7]. The status of the surgical margin is an important factor affecting patient prognosis. Further resection to achieve a negative margin (R0) for pCCA has been shown to significantly improve the prognosis of patients with residual margin invasive cancer (R1) [8–10]. However, whether further resection should be performed in patients with pCCA precursor lesions without malignant transformation shown on intraoperative frozen pathology at the resection margin is undetermined [11–13].

Intraepithelial neoplasia is a common precancerous lesion that can occur in various organs and systems [14–16]. Biliary intraepithelial neoplasia (BilIN) is recognized as a crucial stage in the development of carcinoma [17–19], and it is classified as high-grade (HG, including carcinoma in situ) or low-grade (LG) according to the degree of dysplasia in the bile duct [20]. A series of processes is required for the development of invasive cancer, such as the malignant transformations of bile ducts [21]. However, the time required for these processes to occur as well as the mechanisms of these processes are still unclear based on current research findings. It is also unclear whether the presence of residual BilIN at the surgical margin of pCCA has an impact on survival and tumor recurrence. BilIN is typically diagnosed through intraoperative frozen sections and cannot be macroscopically detected by surgeons [22, 23]. Additional resection of the bile duct containing BilIN can be difficult and increases the rate of surgical complications when hepatectomy and even pancreaticoduodenectomy (PD) are needed, which creates a dilemma for surgeons during the operation. Existing research on this topic usually involves small size samples, and the conclusions are still controversial.

To address this controversy and knowledge gap, a retrospective cohort study on pCCA patients who underwent curative resection in two tertiary hospitals was conducted. The aim of this study was to investigate the impact of residual BilIN detected on intraoperative frozen sections at the surgical margins on the prognosis of patients. This investigation aimed to provide an answer to the question of whether additional resection should be performed in these patients, thus offering more evidence to assist surgeons in intraoperative decision-making.

## Methods

### Study cohort

**Fig. 1:**
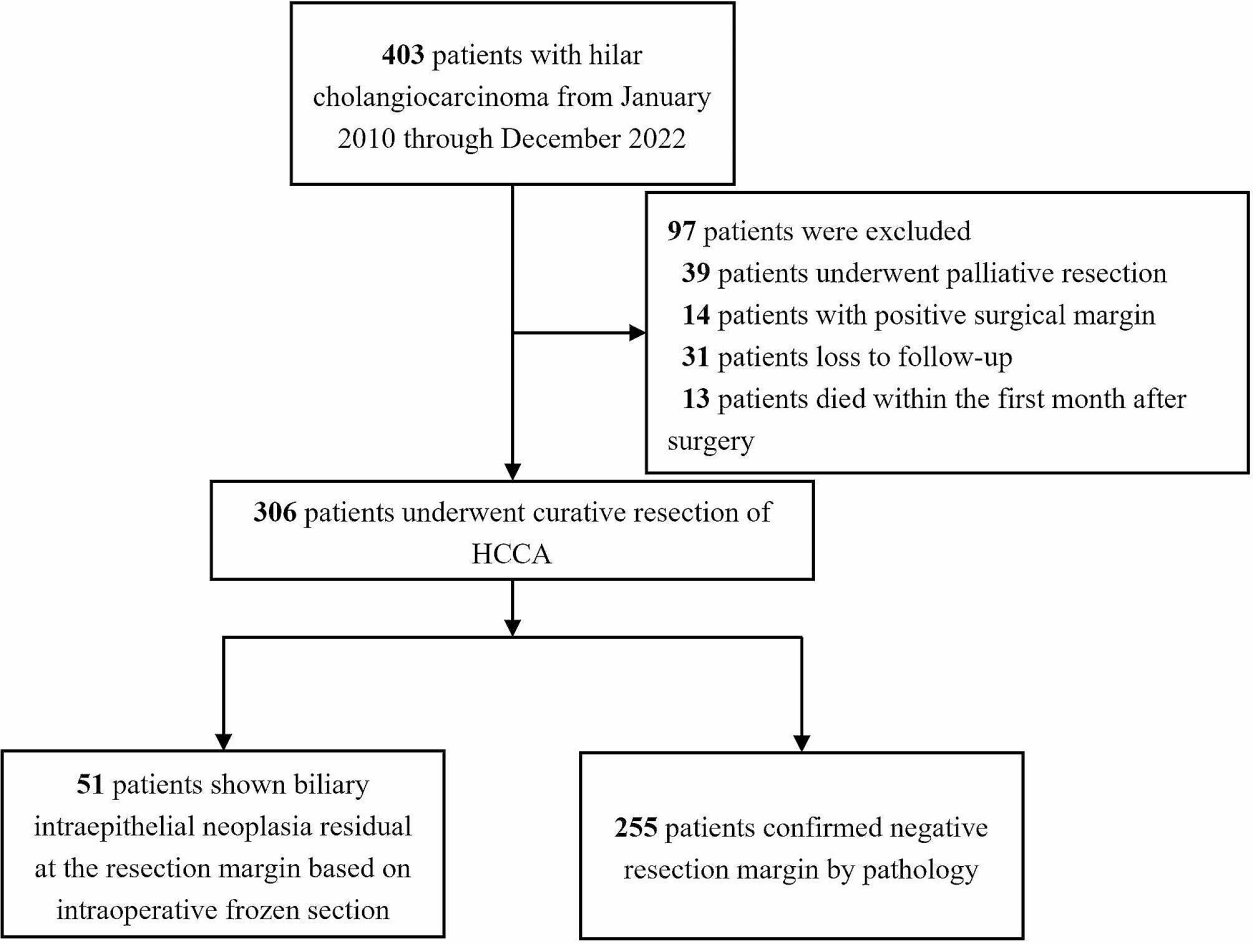
Flow chart of the selection process for perihilar cholangiocarcinoma patients included in the final analysis

This retrospective study cohort included patients from two tertiary hospitals: The First Affiliated Hospital of the Army Medical University and the Sichuan Provincial People’s Hospital. A total of 403 patients diagnosed with pCCA between January 2010 and December 2022 were included, of whom 306 (75.93%) patients underwent curative surgical resection (Fig. 1). The exclusion criteria were palliative resection, confirmed residual invasive cancer at surgical margins, death within 30 days of surgery, and loss to follow-up after surgery. This retrospective analysis complied with the standards of the Declaration of Helsinki. This study was approved by the Ethics Committee of Southwest hospital, the first affiliated hospital of Army military Medical University (No. KY2021129).

### Data collection

Patient data were retrospectively collected from the electronic medical record systems of the two participating hospitals, including demographic characteristics, laboratory variables, operative variables and pathological variables. Laboratory variables were measured in blood samples collected from patients within 3 days before surgery for liver function, which included alanine aminotransferase (ALT), aspartate transaminase (AST), albumin (ALB), total bilirubin (TB), alkaline phosphatase (ALP), glutamyltransferase (GGT), and tumor markers, including carcinoembryonic antigen (CEA), alpha fetoprotein (AFP), carbohydrate antigen 19 − 9 (CA19-9), CA125, and CA24-2. The original images of intraoperative frozen pathology and the final pathological examination were evaluated, and all histopathological sections were reviewed by two experienced biliary pathologists who were blinded to the clinical information. The Bismuth‒Corlette classification of pCCA was determined using the maximum observation method [24].

### Pathological assessment

**Fig. 2:**
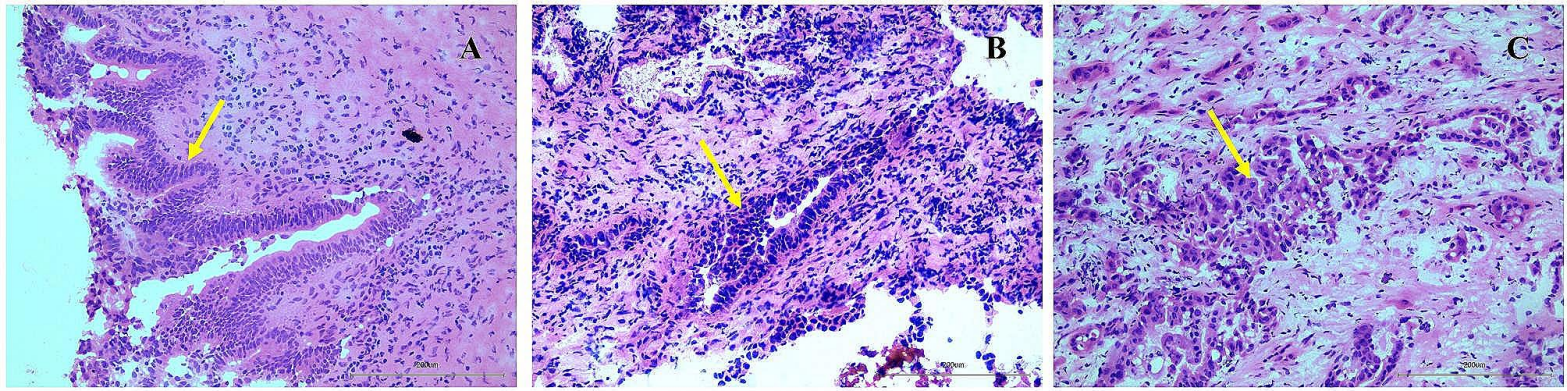
Pathology assessment of the resection margin of the bile duct (HE). **(A)** Low-grade BilIN; **(B)** High-grade BilIN; **(C)** invasive cancer. BilIN, biliary intraepithelial neoplasia

Evaluation of intraoperative frozen pathology results was based on the 5th Edition of the World Health Organization (WHO) Guidelines published in 2019 [20]. Resection margin BilIN was classified as LG and HG based on the degree of dysplasia (flat or micropapillary configuration, loss of cellular polarity and nuclear pseudostratification) of the biliary epithelial cells. The obvious loss of cell polarity is used as the diagnostic criterion for distinguishing HG from LG BilIN. [25] It was also classified according to location at the distal bile duct margin or proximal bile duct. The R0 margin in this study was the absence of residual invasive tumor or BilIN of pCCA at any margin, and R1 resection was defined as the presence of residual invasive cancer of pCCA at resection margins (Fig. 2).

### Treatment and follow-up

All patients underwent preoperative CT or MRI examination and were considered for surgical resection of pCCA. According to both Chinese and Japanese guidelines, we adopted the following criteria of unresectability tumor [26]: organ or peritoneum metastasis, distant lymph node metastasis or locally advanced tumor (vascular involvement or extended bile duct invasion). Curative surgical resection procedures included segmentectomy, hemihepatectomy or trisectionectomy based on the extent of the tumor, with complete resection of the hilar bile duct system and gallbladder and clearance of lymph nodes in the hilum. For patients with Bismuth type I pCCA, if applicable, only local resection at the hepatic hilum and lymph node clearance were performed. Only patients with R1 resection and palliative treatment received adjuvant therapy, while patients with absolute negative margins and those with BilIN at the margins did not undergo adjuvant treatment. After hospital discharge, all patients were closely followed up in outpatient clinics. Basic measurements (liver function, routine blood counts, and abdominal ultrasound) were performed. For patients when tumor recurrence was suspected, further examination of serum tumor markers, CT, MRI and US were conducted for confirmation. Overall survival (OS) and recurrence-free survival (RFS) were calculated from the date of surgery.

### Statistical analysis

Categorical variables are reported as the number of cases and percentage, and continuous variables are expressed as the median and interquartile range (IQR). Survival curves were created using the Kaplan–Meier method, and differences were analyzed using the log-rank test. To evaluate prognostic factors of radical resection, multivariate Cox regression and forest plots were used. The potential risk factors suggested by previous studies and residual BilIN at the surgical margin were included in the final Cox regression model [27]. The selection for subgroup analysis was based on BilIN grading and the results of Cox regression. Based on discernible intraoperative characteristics, further analysis was conducted on whether the presence of BilIN warrants additional resection in cases with risk factors. All statistical analyses were performed using SPSS software version 27.0 (IBM Corp., Armonk, NY, USA) and R software (version 4.2.2 http://www.r-project.org/). All P values were two-sided, and *P* < 0.05 indicated statistical significance. Additionally, a 95% confidence interval was used for assessing the difference.

## Results

### Demographics

Of 403 patients who were diagnosed with pCCA during the study period, 306 *(75.93%)* patients who underwent curative resection surgery were ultimately enrolled. The mean follow-up from surgical resection to the last follow-up visit was 31.6 ± 29.6 months (range: 5-151 months). Among 306 patients, 51 (16.67%) were showed resection margin BilIN by intraoperative frozen section pathology, and subsequently confirmed by postoperative pathology. 255 (83.33%) patients showed absolute R0 margins in both intraoperative and postoperative pathology. The demographic and clinical data of all patients are presented in Table 1.

**Table 1:** Clinical characteristics of included patients

Variable	All patients (*n* = 306)	R0 Resection (*n* = 255)	RM BilIN (*n* = 51)	*P* value
*Demography*				
Sex/male	175 (57.2)	145 (56.9)	30 (58.8)	0.796
Age (years)	59 (50–66)	58 (50–66)	60 (54–68)	0.178
BMI	22.2 (20.2–24.0)	22.3 (20.3–24.0)	22.0 (19.9–24.3)	0.588
Smoking	100 (32.7)	81 (31.8)	19 (37.3)	0.445
Drinking	85 (27.8)	68 (26.7)	17 (33.3)	0.332
ASA ≥ 2	180 (58.8)	151 (59.2)	29 (56.9)	0.755
*Preoperative data*				
ALT (IU/L)	112.5 (61.7-227.9)	108.6 (61.9-216.5)	134.5 (60.2-260.5)	0.541
AST (IU/L)	97.2 (61.0-183.3)	97.2 (61.7-182.2)	91.4 (54.9-197.7)	0.593
ALP (IU/L)	454.0 (294.0-708.0)	454.0 (295.6-705.5)	452.5 (279.3-741.5)	0.920
GGT (IU/L)	549.0 (245.0-940.5)	550.0 (246.7–952.0)	548.0 (234.0-845.4)	0.520
Total bilirubin (mol/L)	196.0 (79.2-293.5)	201.1 (83.1-294.4)	184.1 (55.1-290.6)	0.997
Albumin (g/L)	38.0 (34.6–40.6)	38.0 (34.3–40.4)	39.2 (35.6–41.8)	0.084
CA19-9 (U/ml)	204.87 (83.2-504.7)	205.3 (89.8-504.7)	173.5 (49.6-611.1)	0.177
CA125 (U/ml)	15.9 (9.3–27.2)	16.2 (9.4–27.4)	15.3 (8.7–23.4)	0.618
CA24-2 (U/ml)	18.5 (7.3–65.4)	19.8 (7.3–61.9)	14.8 (7.1–70.9)	0.887
CEA (ug/L)	2.8 (1.8–4.3)	2.8 (1.8–4.3)	2.6 (1.5–3.8)	0.439
HBV positive	20 (6.5)	17 (6.7)	3 (5.9)	0.836
Preoperative biliary drainage	112 (36.6)	90 (35.3)	22 (43.1)	0.288
*Operation features*				
Operation duration (min)	499.0 (391.5-601.3)	495.0 (390.0-600.0)	516.0 (392.0-603.0)	0.940
Intraoperative blood transfusion	174 (56.9)	140 (54.9)	34 (66.7)	0.121
Portal vein invasion	100 (32.7)	81 (31.8)	19 (37.3)	0.445
Hepatic artery invasion	81 (26.5)	67 (26.3)	14 (27.5)	0.862
Large-scale hepatectomy	176 (57.5)	149 (58.4)	27 (52.9)	0.469
*Pathological features*				
Positive lymph nodes	117 (38.2)	98 (38.4)	19 (37.3)	0.875
Perineural invasion	140 (45.8)	115 (45.1)	25 (49.0)	0.608
Microvascular invasion	37 (12.1)	31 (12.2)	6 (11.8)	0.937
Tumor size (cm)	2.0 (2.5–3.5)	2.0 (2.5–3.8)	3.0 (2.0-3.1)	0.917
Poor differentiation	67 (21.9)	58 (22.7)	9 (17.6)	0.422
Bismuth-Corlette classification				0.363
type I and type II	86 (28.1)	69 (27.1)	17 (33.3)	
type III and type IV	220 (71.9)	186 (72.9)	34 (66.7)	

Classification variables are presented as frequency and percentage, n (%); continuous variables are presented as median (IQR, interquartile range); BMI, body mass index; ASA, American Society of Anesthesiologists classification; ALT, alanine transaminase; AST, Aspartate aminotransferase; ALP, alkaline phosphatase; GGT, glutamyltransferase; HBV, Hepatitis B virus; RM BilIN, resection margin involved biliary intraepithelial neoplasia

### Characteristics of patients with BilIN

Among the 51 pCCA patients with BilIN margins, the lesions were distributed by location as follows: distal bile duct—19 patients (37.3%), left hepatic ducts—17 patients (33.3%), and right hepatic ducts—15 patients (29.4%). Intraoperative frozen pathology showed that 42 patients (82.4%) had LG lesions, while 9 patients (17.6%) had HG lesions (Table 2).

**Table 2:** Patients with residual biliary intraepithelial neoplasia at the surgical margin (*n* = 51)

	Classify	*n* (%)
Location	Distal bile duct	19 (37.3)
	Left hepatic duct	17 (33.3)
	Right hepatic duct	15 (29.4)
Degree	Low-grade	42(82.4)
	High-grade	9 (17.6)

### Survival analysis

**Fig. 3:**
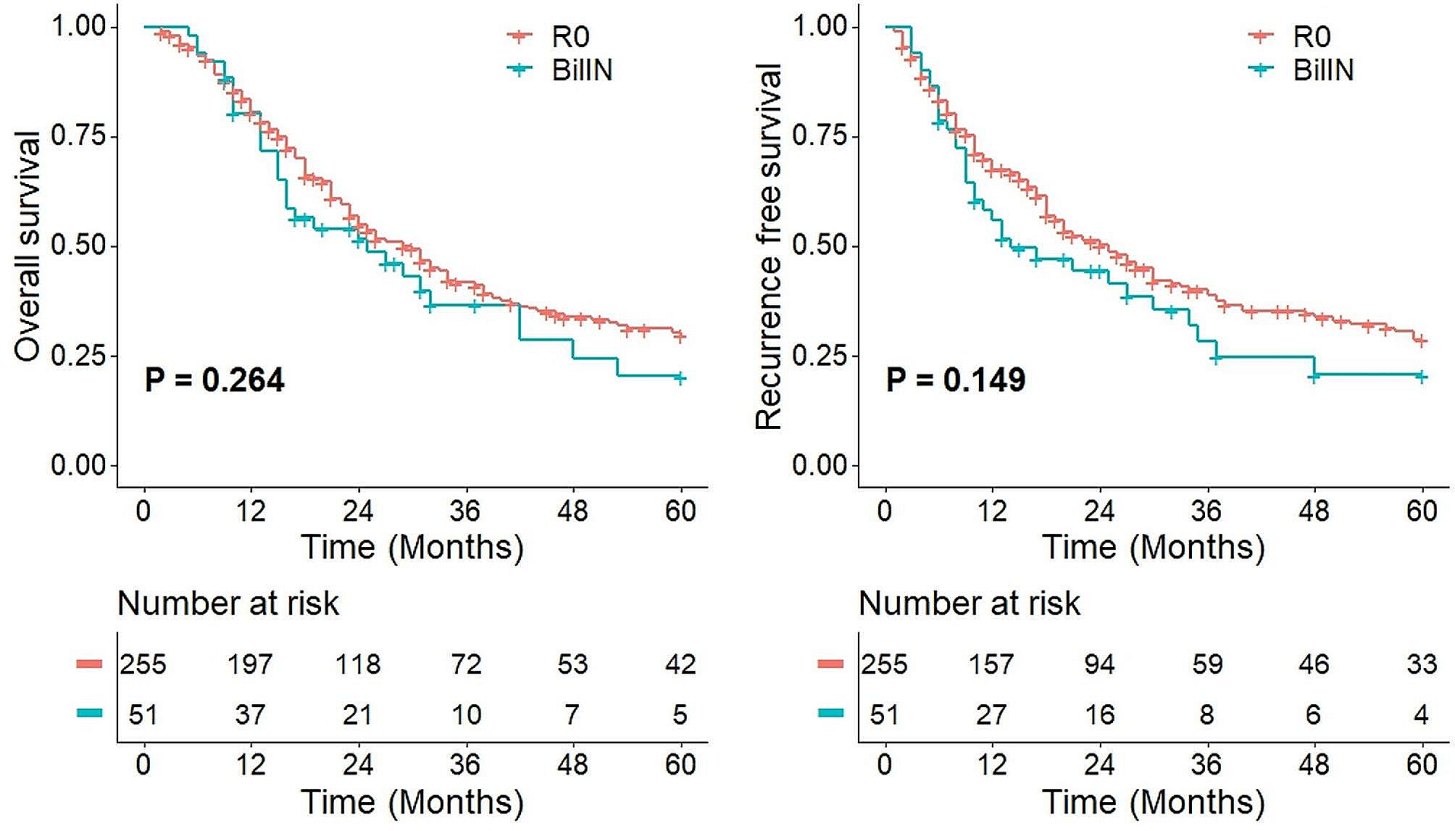
Overall survival and recurrence-free survival in all participating patients

The 1-, 3-, and 5-year OS rates for the entire cohort were 82.8%, 56.3%, and 28.8%, respectively, and the RFS rates were 67.7%, 50.0%, and 27.3%, respectively. The 1-, 3-, and 5-year OS rates for patients with R0 margins were 83.3%, 56.8%, and 30.4%, respectively, and the RFS rates were 69.7%, 51.2%, and 28.7%, respectively. The 1-, 3-, and 5-year OS rates for patients with residual BilIN margins were 80.2%, 54.0%, and 20.3%, respectively, and the RFS rates were 58.2%, 44.5%, and 20.6%, respectively. There were no significant differences in long-term survival or recurrence-free survival between these two groups of patients (*P* = 0.264 and *P* = 0.149, respectively) (Fig. 3).

### Cox regression defines independent risk factors

**Fig. 4:**
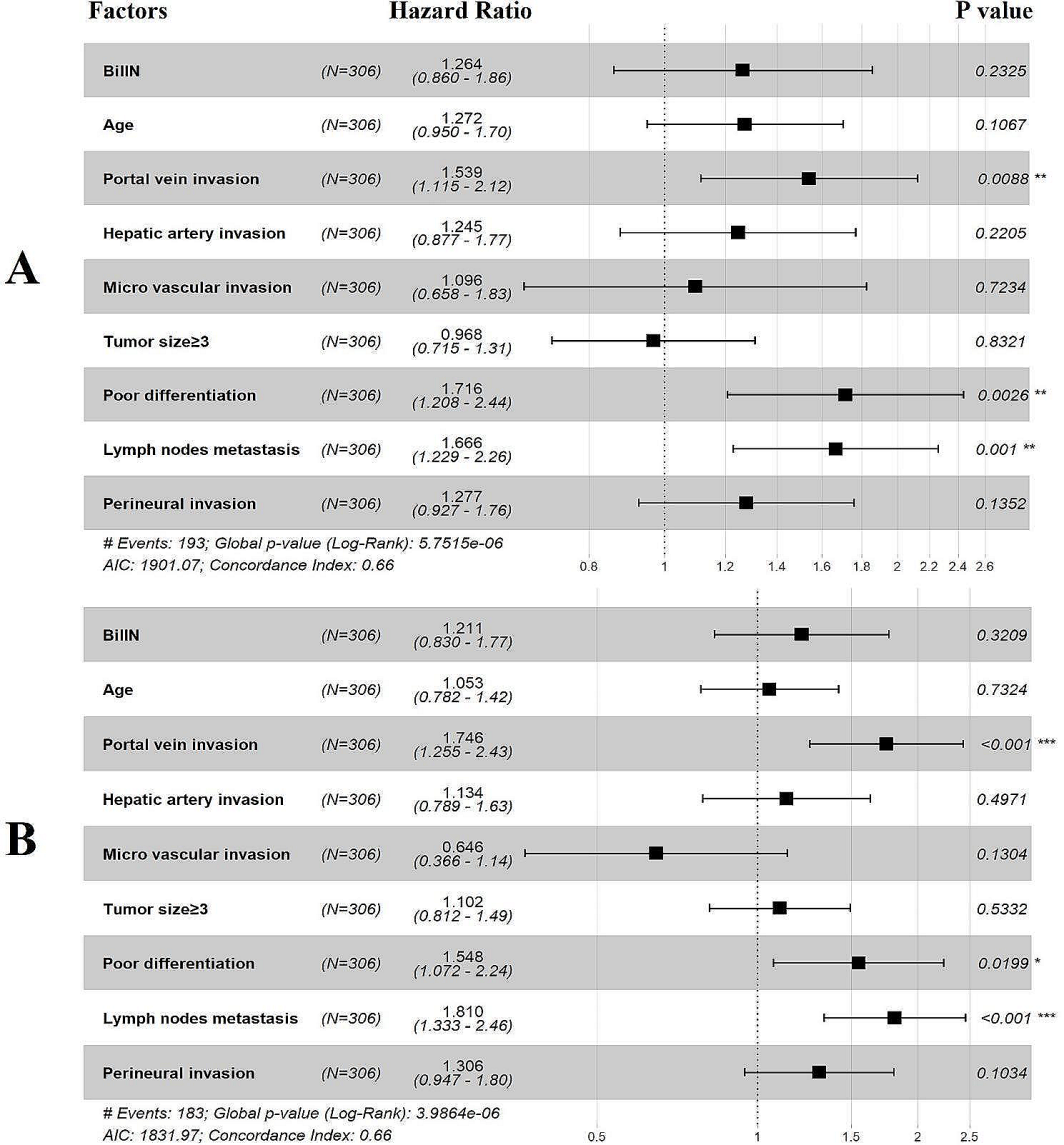
Multivariate Cox regression clinicopathological factors associated with OS **(A)** and RFS **(B)** in the entire patient cohort. BilIN, biliary intraepithelial neoplasia

Multivariate Cox regression analysis was conducted to define the prognostic risk factors for pCCA for all the included patients, and 9 potential variables were included. The results of Cox regression are presented in the form of a forest plot (Fig. 4). The independent risk factors that significantly affected OS included portal vein invasion (*P* = 0.009, HR = 1.539), poor differentiation (*P* = 0.003, HR = 1.716) and lymph node metastasis (*P* = 0.001, HR = 1.666). Independent risk factors that significantly affected RFS included portal vein invasion (*P* < 0.001, HR = 1.746), poor differentiation (*P* = 0.019, HR = 1.548) and lymph node metastasis (*P* < 0.001, HR = 1.810).

### Subgroup analysis

**Fig. 5:**
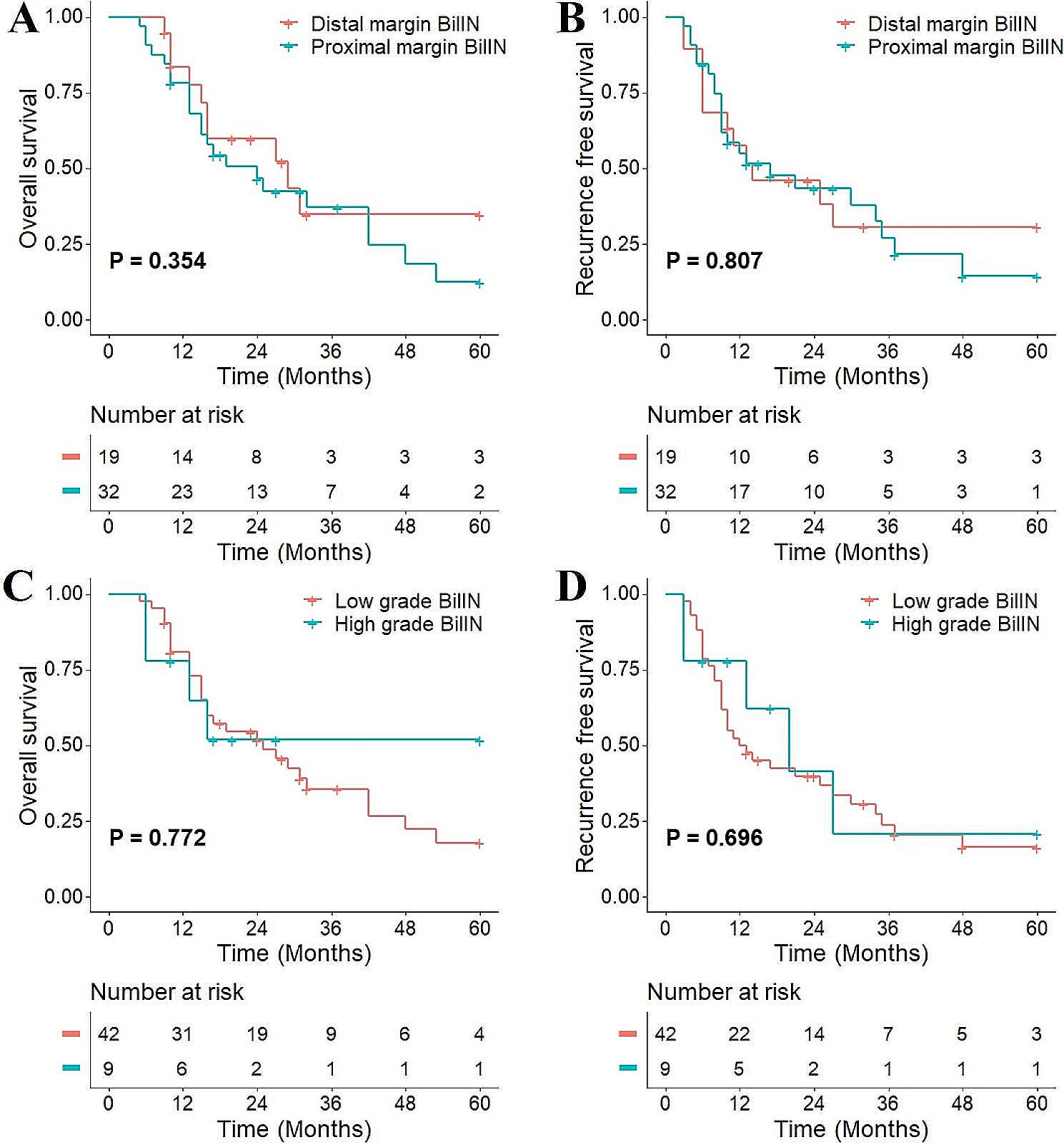
Subgroup analysis based on BilIN location and grade. **(A)** OS in patients based on resection margin location; **(B)** RFS in patients based on resection margin location; **(C)** OS in patients based on BilIN grade; **(D)** RFS in patients based on BilIN grade. BilIN, biliary intraepithelial neoplasia; OS, overall survival; RFS, recurrence-free survival

**Fig. 6:**
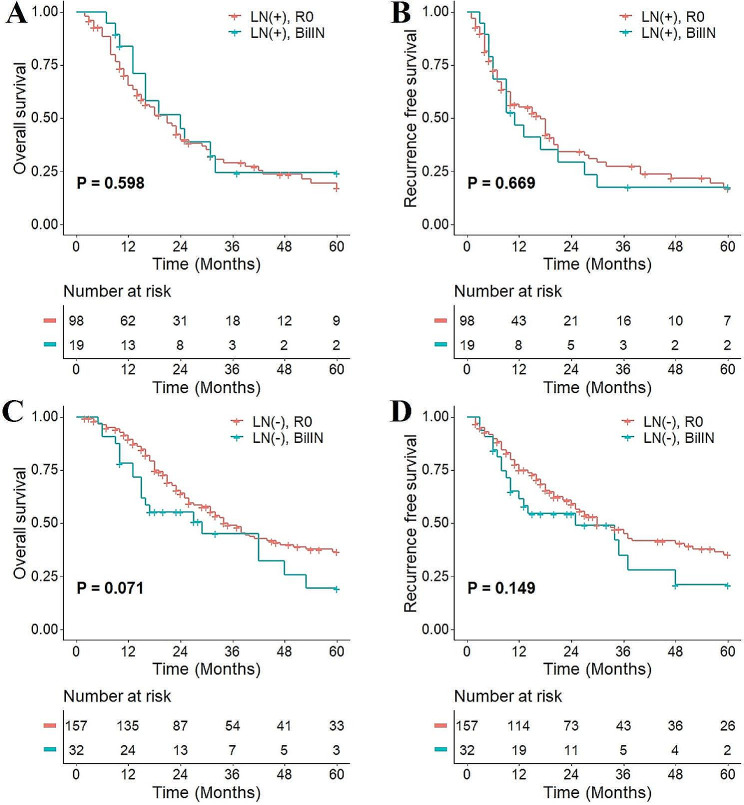
Subgroup analysis based on lymph node (LN) metastasis status. **(A)** OS in patients without lymph node metastasis; **(B)** RFS in patients without lymph node metastasis; **(C)** OS in patients with lymph node metastasis; **(D)** RFS in patients with lymph node metastasis. BilIN, biliary intraepithelial neoplasia; OS, overall survival; RFS, recurrence-free survival

**Fig. 7:**
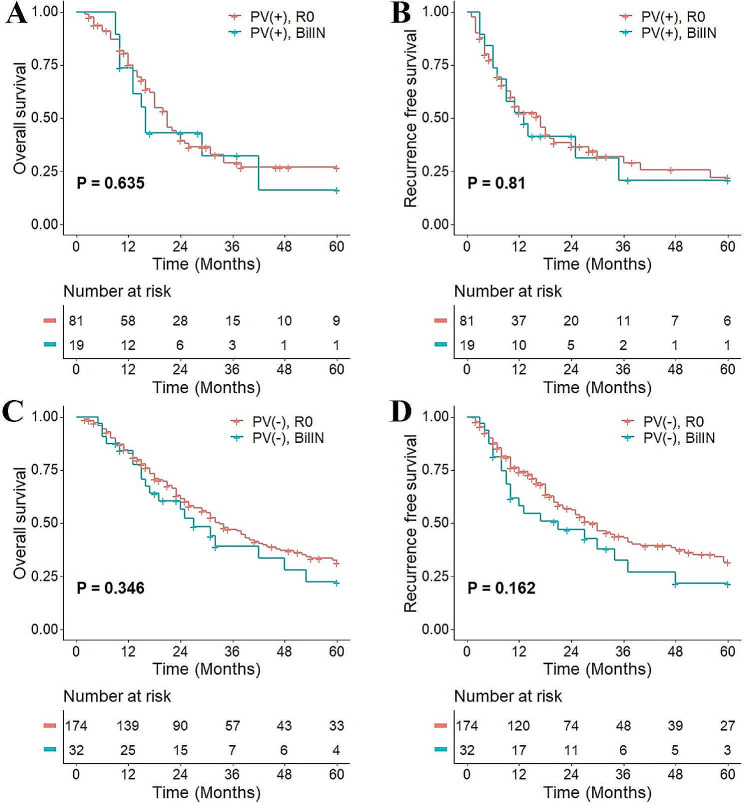
Subgroup analysis based on portal vein (PV) invasion status. **(A)** OS in patients without portal vein invasion; **(B)** RFS in patients without portal vein invasion; **(C)** OS in patients with portal vein invasion; **(D)** RFS in patients with portal vein invasion. BilIN, biliary intraepithelial neoplasia; OS, overall survival; RFS, recurrence-free survival

The impact of residual BilIN at different locations and on different grades on patient prognosis was analyzed. No significant difference was found in patient OS (*P* = 0.354) or RFS (*P* = 0.807) between distal bile duct margin and proximal bile duct involvement (Fig. 5A and B). Among 51 patients, 9 had HG lesions, and no significant difference was found in OS (*P* = 0.772) or RFS (*P* = 0.696) between patients in the LG and HG groups (Fig. 5C and D). Further subgroup analyses were performed based on the presence or absence of lymph node metastasis and portal vein invasion. No significant differences were found in OS and RFS between pCCA patients who had R0 resection margins and residual BilIN at resection margins in the N0 subgroup and N1 subgroup (Fig. 6). Similarly, there was also no significant difference in OS and RFS between pCCA patients with residual BilIN at the resection margin and those with R0 resection margin in the portal vein invasion subgroup (Fig. 7).

## Discussion

Surgical resection has been considered the only curative treatment for pCCA patients, and the status of the surgical margin is an important factor that affects patients’ long-term survival. Residual invasive cancer at resection margins results in poor prognosis, and resection margins to achieve a tumor-free margin are widely recommended [11, 28]. However, there is still controversy over whether further resection is necessary for residual BilIN at the pCCA resection margin. In this retrospective study, we demonstrated that such BilIN at the resection margin is safe for patients undergoing curative resection for pCCA and did not significantly affect their OS or RFS, and no further resection is needed.

The development of intraoperative frozen pathology has given surgeons a new look at surgical resection margins, and residual BilIN without malignant transformation at the margin has been defined and brought into the surgeons’ attention [22, 28, 29]. The 5th Edition of the WHO Guidelines published in 2019 redefined and recommended using intraepithelial neoplasia instead of dysplasia to describe such biliary lesions. Additionally, carcinoma in situ (CIS) has been classified as HG BilIN, and the previous three-tiered grading system has been replaced with a two-tiered system to describe this precancerous lesion [20]. This type of residual lesion is often difficult to identify by visual examination and cannot be clearly classified as R0 or R1 resection. Intraoperative frozen pathology is the only method of identification, although its sensitivity and specificity are lower compared to postoperative pathology. Nonetheless, it remains the best way to determine the extent of resection during intraoperative decision-making. There is still uncertainty whether margin BilIN have a worse prognosis than those with negative margins and whether further resection should be performed for such lesions after intraoperative frozen pathology diagnosis to achieve an absolute negative margin.

The results of several studies have suggested that cholangiocarcinoma patients with residual CIS at resection margins have survival rates similar to those patients after R0 resection, and these patients should not undergo further resection, even though they may have a higher risk for late local recurrence [30, 10, 31, 32]. In contrast, in recent years, scholars in some studies on extrahepatic cholangiocarcinoma (eCCA) proposed that for patients with CIS or HG BilIN in distal bile ducts, further resection should be performed using techniques such as intrapancreatic bile duct resection or even combined PD to achieve an absolute negative margin to the greatest possible extent [33–35, 32, 36]. However, based on our research, we believe that this conclusion may not be applicable to patients with pCCA. First, the results of this study demonstrated that there was no significant difference in long-term survival between patients with residual BilIN without malignant transformation at the resection margin and those with negative margins. Furthermore, compared to eCCA, pCCA is located more proximally in the biliary anatomy, which generally requires the removal of a portion of the liver to achieve curative resection. In our cohort of 306 patients with pCCA, only 16 patients did not undergo liver resection, while 176 patients underwent extensive liver resections (the removal of *3* or more liver segments), which makes achieving a more extensive resection challenging.

Subgroup analysis was performed on residual BilIN at different locations. The results showed that there was no significant difference in long-term survival between patients with residual BilIN at the distal bile duct resection margin and those with residual BilIN at the proximal bile duct resection margin. Further resection not only has no significant long-term outcome benefits but also subjects patients to greater surgical risks. Different surgical strategies are needed for further resection at different locations. While expanding the extent of PD is necessary in patients with residual distal bile ducts, extremely extensive hepatopancreatoduodenectomy (HPD) poses increased *complications* to patients. A systematic review showed a 12% mortality rate three months after HPD in biliary cancer patients, which is unacceptable compared to the 2% mortality rate in our center for routine pCCA surgery [36–38]. In patients with residual disease at the proximal bile duct, especially those with Bismuth III/IV pCCA, half liver resection or trisectionectomy may have been performed at the time of pathological examination [39–40]. It is technically extremely difficult to dissect any additional 5 mm of proximal bile duct in the remaining liver. On the basis of having already excised a sufficiently large portion of the liver, further resection is challenging to ensure a balance between the extent of resection and the volume of the remaining liver. Therefore, opting for overly aggressive surgical approaches in pursuit of absolute negative margins is not recommended [41–42]. In this study cohort, although there was no statistically significant difference, patients in the BilIN group exhibited a lower 5-year survival rate compared to the R0 group. This highlights the need to explore non-surgical approaches to improve outcomes for such patients. We believe that closer follow-up and personalized adjuvant treatment plans would be more reasonable approaches for such patients, rather than additional resection. Prior to 2019, adjuvant therapy was not considered beneficial for patients with R0 resected CCA [43]. However, with the initiation of several prospective clinical trials, routine adjuvant therapy post-surgery has gained increasing attention from surgeons [44]. The 2023 NCCN guidelines have recommended adjuvant therapy for patients with R0 resection [45]. Although we have also started to administer postoperative chemotherapy to pCCA patients, due to limited follow-up time and sample size, none of the patients included in our study received adjuvant therapy. Further research is needed to determine the benefits of adjuvant therapy for patients with margin-positive BilIN, which would be a meaningful question worth exploring further and research in this field is currently lacking.

Multivariate Cox regression analysis was conducted in our study to identify the risk factors affecting the long-term survival of patients after pCCA curative resection, and BilIN without malignant transformation at the pCCA surgical margin was not an independent risk factor affecting the prognosis of patients. The results suggested that lymph node metastasis, portal vein invasion, and poor differentiation were independent risk factors affecting OS and RFS. As lymph node metastasis and portal vein invasion can be determined by surgery and intraoperative frozen pathology, poor tumor differentiation can only be accurately determined by postoperative pathology [22, 29, 30]. Higuchi et al. proposed that achieving an R0 margin was associated with improved survival in early-stage N0M0 patients compared to those who had residual HG BilIN at the margins without malignant transformation [36]. A retrospective study was conducted to evaluate the impact of margin status with different risk factors on patient prognosis. [46] It was suggested that tumor infiltration around the margins may have a greater impact on prognosis compared to ductal infiltration. Therefore, we conducted another subgroup analysis, which included the presence or absence of lymph node metastasis and portal vein invasion. The results showed that regardless of the presence of lymph node metastasis or portal vein invasion, residual BilIN without malignant transformation at resection margins did not have any significant impact on patient prognosis. Notably, the main difference between our study and others is the inclusion of LG BilIN lesions in our patient cohort.

Although residual BilIN at the surgical margins can undergo malignant transformation, these lesions remain in the body for a long period, and related studies have indicated that additional carcinogenic events are needed to promote invasion and progression [19, 21]. Compared to the highly invasive nature of pCCA, we considered that this slow process can contribute little to pCCA recurrence, and there is no sufficient evidence to show that these lesions affect patient survival. For patients with margin-positive BilIN, we believe that closer follow-up and monitoring of tumor recurrence should be conducted. Furthermore, frozen section analysis is the only method to determine the BilIN margins during surgery, and it can be affected by factors such as biliary inflammation, drainage techniques, and surgical procedures, making it difficult to recognize epithelial lesions such as dysplasia [28]. Similar studies on pancreatic tumors have also shown that precursor lesions at surgical margins are not associated with OS after surgery, and additional resection up to total pancreatectomy did not provide any additional survival benefit [47].

This study still has several limitations. First, due to the retrospective study design, there could be selection bias and reporting bias. Second, although this study included data from two centers, the low incidence of resectable pCCA and residual BilIN margins led to a relatively small sample size. Third, some studies have suggested that adjuvant therapy for perihilar cholangiocarcinoma confer a favorable prognosis, but it is not used in the participating centers, further research is needed to verify whether the presence of BilIN at the resection margin affects the survival of patients receiving adjuvant therapy.Fourth, due to the limited follow-up duration, a complete picture of the influence of residual BilIN at the resection margin without malignant transformation on the long-term prognosis of patients may not have been obtained, and further research with a longer follow-up is needed.

**Fig. 8:**
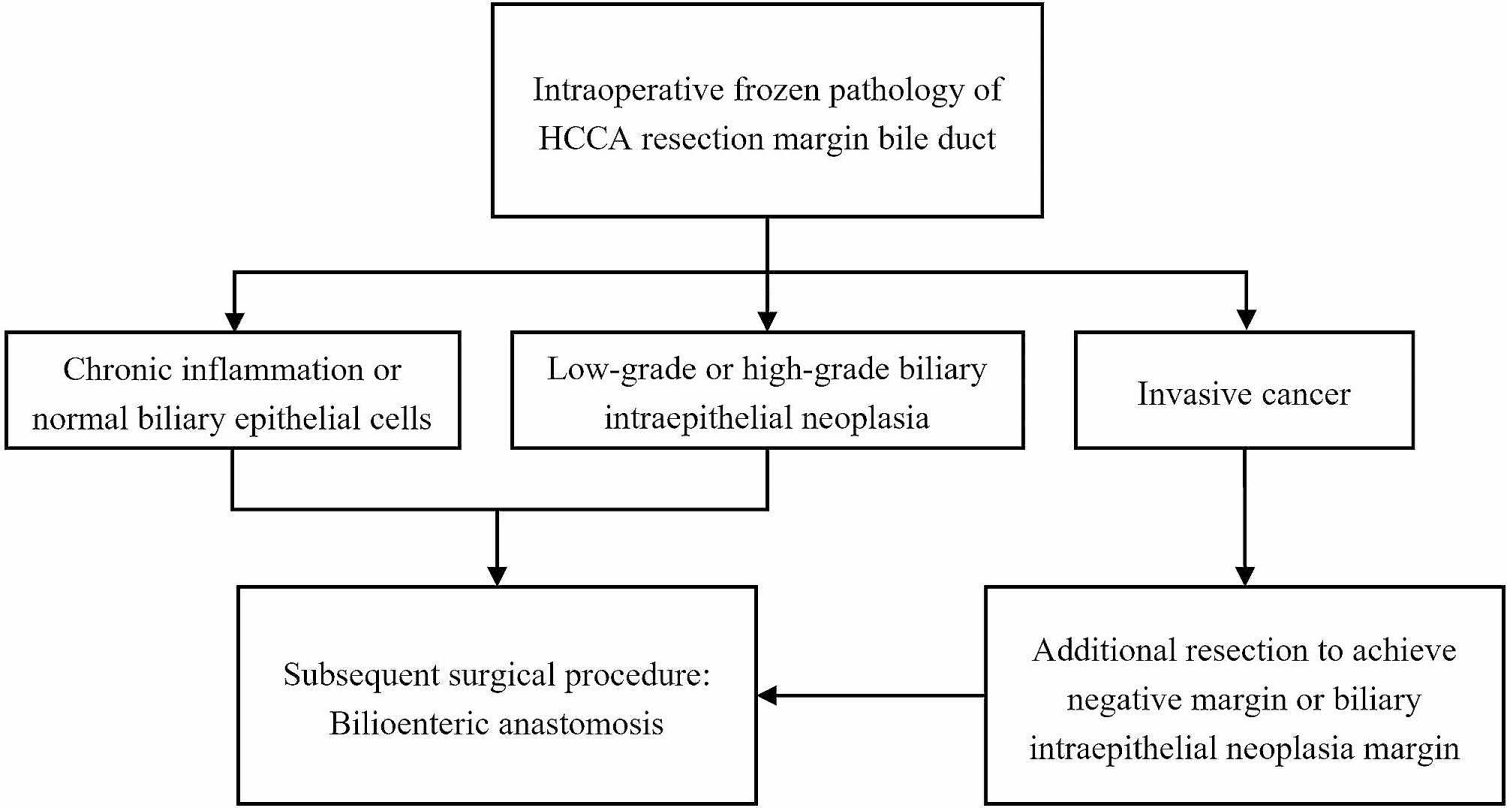
Flow chart of recommended surgical treatment options based on intraoperative frozen pathology

In summary, residual BilIN at the surgical margin without malignant transformation does not significantly affect the survival of pCCA patients. In those patients, regardless of whether the lesions were HG or LG and regardless of where they were located, We do not recommend extensive surgical resection to pursue absolute negative margin. (Fig. 8) The findings will help to improve surgeons’ decision-making during surgery and help to avoid unnecessary additional resections.

## Data Availability

The data that support the findings of this study are available from [Prof. Chengcheng Zhang, E-mail: zccszcg@163.com], upon reasonable request.
